# Macrophage autophagy protects mice from cerium oxide nanoparticle-induced lung fibrosis

**DOI:** 10.1186/s12989-021-00398-y

**Published:** 2021-02-01

**Authors:** Balasubramanyam Annangi, Zhuyi Lu, Jonathan Bruniaux, Audrey Ridoux, Vanessa Marques da Silva, Delphine Vantelon, Jorge Boczkowski, Sophie Lanone

**Affiliations:** 1 Univ Paris Est Creteil, INSERM, IMRB, F-94010 Creteil, France; 2 Synchrotron SOLEIL, L’orme des merisiers, St Aubin, BP 48, 31192 Gif sur Yvette, Cedex France; 3 AP-HP, Hopital Henri Mondor, Service Pneumologie, F-94010 Creteil, France

**Keywords:** Alveolar fibrosis - autophagy - macrophage polarization, Nanoparticle

## Abstract

**Background:**

Cerium (Ce) is a rare earth element, rapidly oxidizing to form CeO_2_, and currently used in numerous commercial applications, especially as nanoparticles (NP). The potential health effects of Ce remain uncertain, but literature indicates the development of rare earth pneumoconiosis accompanied with granuloma formation, interstitial fibrosis and inflammation. The exact underlying mechanisms are not yet completely understood, and we propose that autophagy could be an interesting target to study, particularly in macrophages. Therefore, the objective of our study was to investigate the role of macrophagic autophagy after pulmonary exposure to CeO_2_ NP in mice. Mice lacking the early autophagy gene *Atg5* in their myeloid lineage and their wildtype counterparts were exposed to CeO_2_ NP by single oropharyngeal administration and sacrificed up to 1 month after. At that time, lung remodeling was thoroughly characterized (inflammatory cells infiltration, expression of fibrotic markers such as αSMA, TGFβ1, total and type I and III collagen deposition), as well as macrophage infiltration (quantification and M1/M2 phenotype).

**Results:**

Such pulmonary exposure to CeO_2_ NP induces a progressive and dose-dependent lung fibrosis in the bronchiolar and alveolar walls, together with the activation of autophagy. Blockage of macrophagic autophagy protects from alveolar but not bronchiolar fibrosis, via the modulation of macrophage polarization towards M2 phenotype.

**Conclusion:**

In conclusion, our findings bring novel insight on the role of macrophagic autophagy in lung fibrogenesis, and add to the current awareness of pulmonary macrophages as important players in the disease.

**Supplementary Information:**

The online version contains supplementary material available at 10.1186/s12989-021-00398-y.

## Background

Until recent years, rare earth elements (REE) received limited attention from environmental and public health researchers. In the last two decades, they have however undergone a fantastic boost in their technological and industrial utilization, which is accompanied by concerns regarding emissions and potential human exposures. One of the main REE currently used in numerous commercial applications is Cerium (Ce), as Ce is very reactive and is a strong oxidizing agent, rapidly oxidizing when in contact with oxygen to form CeO_2_. Ce is for example used in flat screen display, alloys, petroleum refining (cracking catalyst), ceramics, glass additives, phosphors, or polishing compounds for glass mirrors, plate glass, television tubes, ophthalmic lenses, precision optics, electronic wafers [1]. CeO_2_ is also used as additive in cigarettes, and CeO_2_ nanoparticles (NP) are predominantly used as diesel fuel additive to increase fuel combustion efficiency [2]. Because of these already numerous current and foreseen applications, CeO_2_ NP might enter the environment through disposal of consumer and industrial products, and this is accompanied by a growing concern for human health.

The potential health effects of Ce remain uncertain, but RE pneumoconiosis accompanied with granuloma and interstitial fibrosis has been reported in workers exposed to asbestos and RE dusts, together with the presence of Ce-containing particles in macrophages from bronchoalveolar lavage (BAL) and in interstitial macrophages [3, 4]. Experimental studies conducted in mice or rats have shown that animal exposure to CeO_2_ NP leads to the development of pulmonary fibrosis, accompanied by a persistent pulmonary inflammation and the presence of oxidative stress [1, 5–9]. The exact underlying mechanisms are not yet completely understood, and we propose that autophagy could be an interesting target to study.

Autophagy is a physiological process mainly responsible for the recycling of damaged cellular organelles and/or macromolecules into simpler forms for cell survival. The resulting degradation products are recycled to maintain nutrient and energy homeostasis [10, 11]. Apart from its importance in physiological conditions, numerous studies have suggested that autophagy is regulated under different pathophysiological conditions, in particular in the lung fibrotic process [12, 13]. We have recently demonstrated that macrophagic autophagy can be modulated after exposure to NP [14], which could be of relevance in the context of pulmonary response to Ce exposure given that macrophages are important players in fibrotic disease [15–17].

Therefore, the objective of the present study was to investigate the role of macrophagic autophagy after pulmonary exposure to CeO_2_ NP in mice. To achieve our aim, mice lacking the early autophagy gene *Atg5* in their myeloid lineage and their wildtype counterparts were exposed to CeO_2_ NP by single oropharyngeal administration and sacrificed up to 1 month after. At that time, lung remodeling was thoroughly characterized (inflammatory cells infiltration, expression of fibrotic markers such as αSMA, TGFβ1, total and type I and III collagen deposition), as well as macrophage infiltration (quantification and M1/M2 phenotype).

## Results

### Characterization of pulmonary remodeling and autophagy modulation in response to CeO_2_ NP

**Fig. 1:**
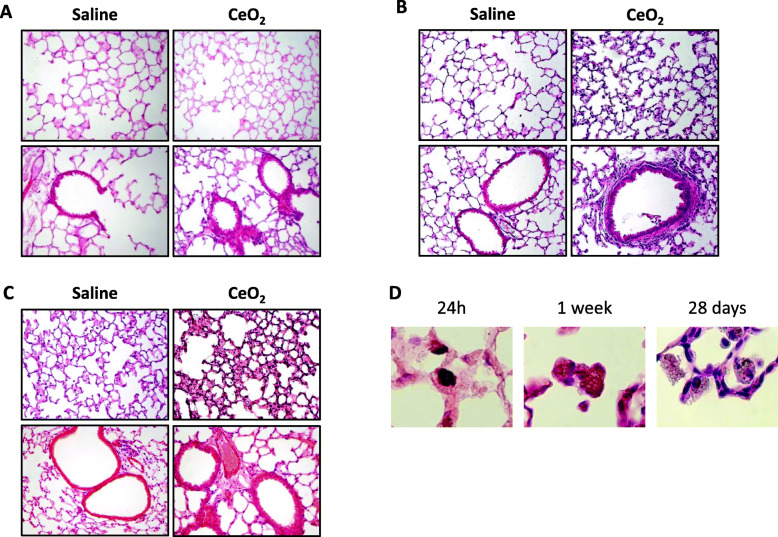
Exposure to CeO_2_ NP induces a progressive lung remodeling. Representative lung tissue sections of C57Bl/6 mice exposed to Saline or CeO_2_ NP and stained with Hematoxylin Eosin (HE) after 1 day (Panel **a**), 7 days (Panel **b**) or 28 days (Panel **c**). Original magnification × 200. Panel **d**: higher magnification of alveolar macrophages with internalized NP

**Fig. 2:**
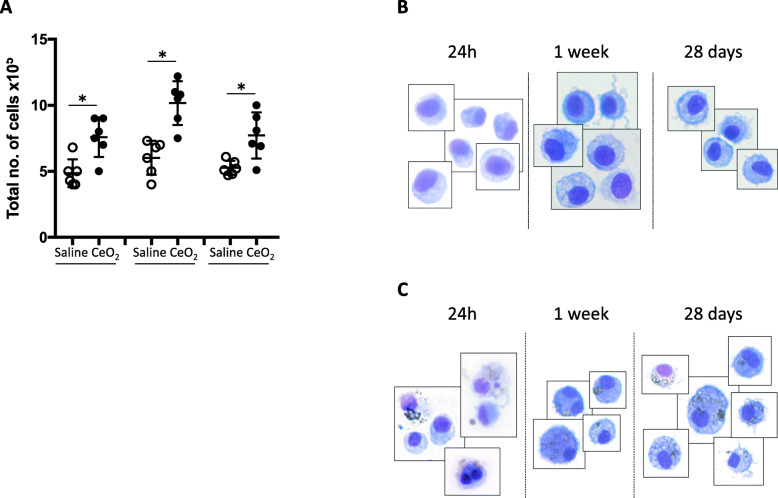
Exposure to CeO_2_ NP induces an increase in BAL cellularity. Quantification of total cell count in BAL fluid (Panel **a**). Each individual circle represents the value obtained from one animal (empty circle: saline exposure – plain circle: CeO_2_ NP-exposure). *p < 0.05. Typical images of BAL macrophages from Saline-exposed (Panel **b**) or CeO_2_ NP-exposed (Panel **c**) mice sacrifice after 24 h, 1 week or 28 days

Exposure of C57Bl/6 mice to a single administration of 50 μg CeO_2_ NP induced a profound lung fibrotic remodeling characterized by a progressive thickening of bronchiolar and alveolar walls. These modifications appeared gradually, starting from 24 h post CeO_2_ NP administration for bronchiolar modifications, and after 1 week for the alveolar ones (Fig. 1a-c). CeO_2_ NP were internalized in macrophages present in the alveolar area (Fig. 1d). This was accompanied by a persistent increased total cell count in the BAL (Fig. 2a). Cell differential analysis identified macrophages as the predominant cell population, with a transient significant increase of neutrophil percentage in BAL from CeO_2_-exposed animal 24 h and 1 week after their initial exposure, but which was no longer present after 1 month (Additional Figure 1). As shown in Fig. 2, CeO_2_ NP were internalized in BAL macrophages at all time points.

**Fig. 3:**
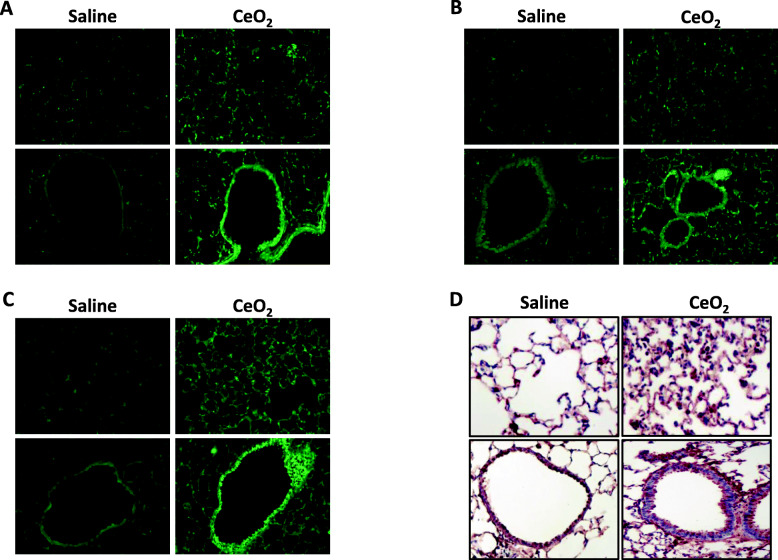
Exposure to CeO_2_ NP induces autophagy. Representative lung tissue sections of GFP-LC3 mice exposed to Saline or CeO_2_ NP after 1 day (Panel **a**), 7 days (Panel **b**) or 28 days (Panel **c**). Original magnification × 200. Panel **d**: representative lung tissue sections of C57Bl/6 mice exposed to Saline or 50 μg CeO_2_ NP and stained, after 28 days, for Atg5. Original magnification × 200

To investigate whether autophagy was induced in our experimental model of lung fibrosis, we utilized GFP-LC3 transgenic mice and exposed them to 50 μg CeO_2_ NP. As shown in Fig. 3, we were able to observe the induction of autophagy in CeO_2_-exposed GFP-LC3 mice, already 24 h after the initial administration, and up to 28 days. This was observed mainly in epithelial cells in bronchiolar regions, and in alveolar regions (Fig. 3). These results were confirmed by the observation of increased levels of Atg5 protein, an early marker of autophagy pathway, in similar regions of CeO_2_-exposed mice (Fig. 3d).

As macrophages are supposedly important players in lung fibrosis [15–18], and as we recently demonstrated the importance of autophagy in macrophagic response to NP [14], we next focus our work on macrophages. The potential of CeO_2_ NP to induce autophagy in macrophages was confirmed by the observed accumulation of GFP-LC3 puncta in peritoneal macrophages obtained from GFP-LC3 mice and exposed to CeO_2_ NP in vitro (Additional Figure 2A), as well as by the induction of Atg5 protein expression in peritoneal macrophages of C57Bl/6 mice exposed to CeO_2_ NP in vitro (Additional Figure 2B). Finally, as autophagy is a highly dynamic process, we addressed the issue of its activation in macrophages exposed to CeO_2_ NP. As shown in Additional Figure 2C, we could observe the colocalization of LC3 staining with that of the lysosomal protein LAMP-1 in CeO_2_ NP-exposed macrophages. Overall, these results indicate that pulmonary exposure to CeO_2_ NP is followed by the activation of autophagy, including in macrophages, which are able to phagocyte CeO_2_ NP.

### Effect of blockage of macrophagic autophagy in response to CeO_2_ NP

In order to decipher the specific role of macrophagic autophagy in the development of lung fibrosis, mice lacking *Atg5* gene in their myeloid lineage (Atg5^flox/flox^ LysM^Cre^ or *Atg5*^*+/−*^) were exposed to CeO_2_ NP. Moreover, to further characterize our model, we performed a dose-response experiment and analyzed the lung remodeling achieved in mice 28 days after exposure to one single administration of either 5 or 50 μg CeO_2_ NP.

**Fig. 4:**
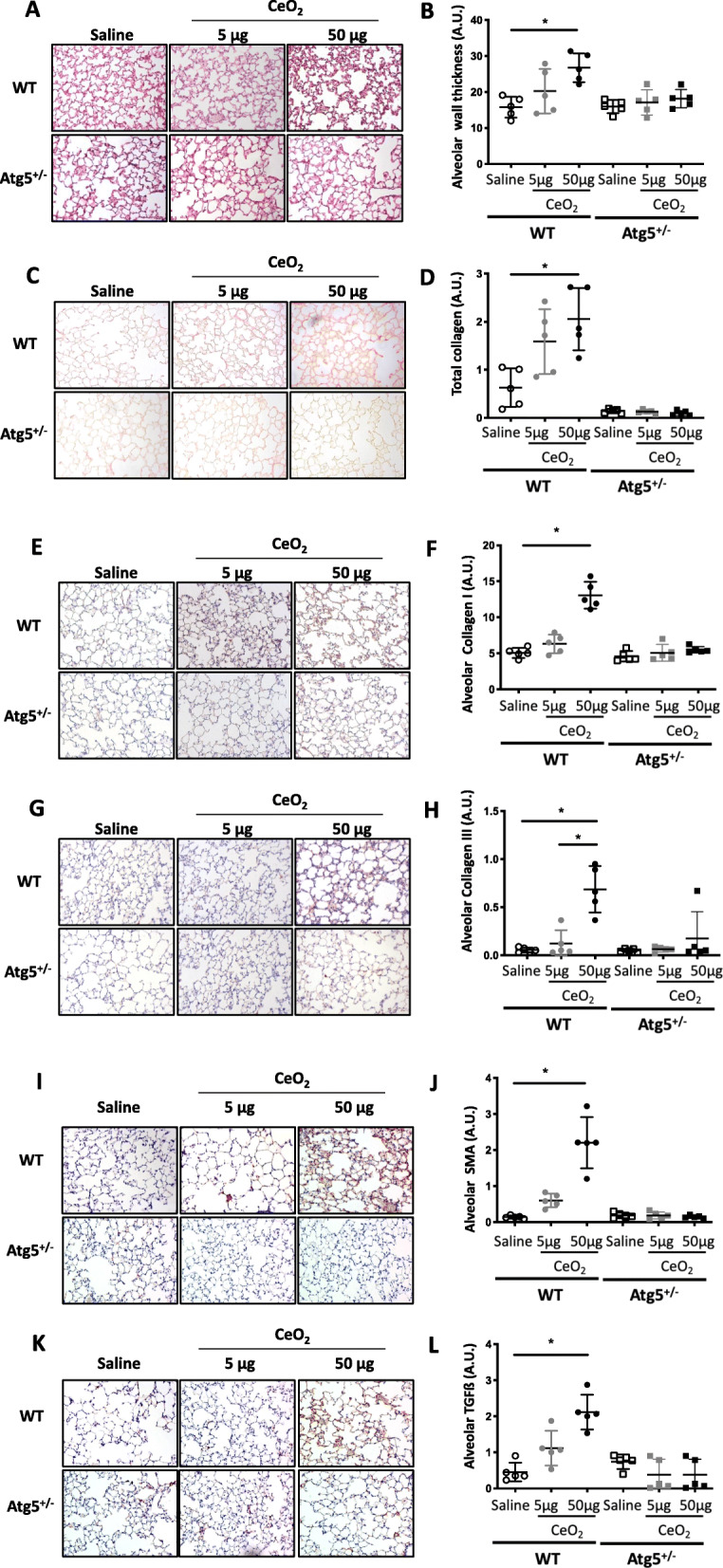
Atg5^+/−^ mice are protected from alveolar remodeling. Representative lung tissue sections of WT and Atg5^+/−^ mice exposed to Saline or CeO_2_ NP (5 or 50 μg, observation at 28 days), after HES (Panel **a** and **b** for quantification), Sirius red (Panel **c** and **d** for quantification), Collagen type I (Panel **e** and **f** for quantification), Collagen type III (Panel **g** and **h** for quantification), αSMA (Panel **i** and **j** for quantification) and TGF-ß (Panel **k** and **l** for quantification). Original magnification × 200. Each individual circle represents the mean value obtained from one animal. Empty circle: saline exposure. Grey circle: 5 μg CeO_2_ NP exposure. Plain circle: 50 μg CeO_2_ NP exposure. *p < 0.05

**Fig. 5:**
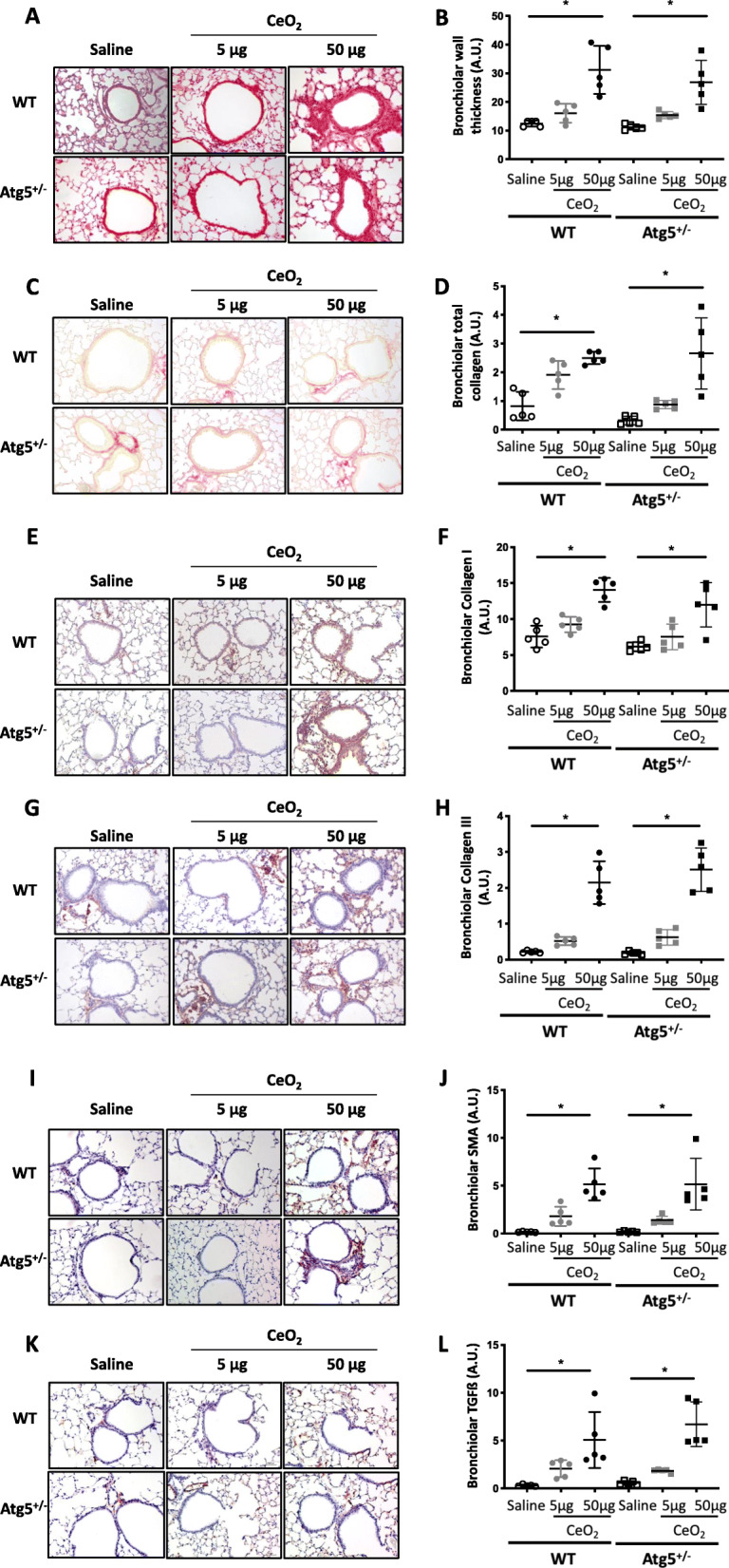
Atg5^+/−^ mice are not protected from bronchiolar remodeling. Representative lung tissue sections of WT and Atg5^+/−^ mice exposed to Saline or CeO_2_ NP (5 or 50 μg, observation at 28 days), after HES (Panel **a** and **b** for quantification), Sirius red (Panel **c** and **d** for quantification), Collagen type I (Panel **e** and **f** for quantification), Collagen type III (Panel **g** and **h** for quantification), αSMA (Panel **i** and **j** for quantification) and TGF-ß (Panel **k** and **l** for quantification). Original magnification × 200. Each individual circle represents the mean value obtained from one animal. Empty circle: saline exposure. Grey circle: 5 μg CeO_2_ NP exposure. Plain circle: 50 μg CeO_2_ NP exposure. *p < 0.05

As shown in Figs. 4 & 5, Atg*5*^*+/−*^ mice do not present any particular lung phenotype in absence of exogenous exposure (Saline condition), and their macrophages do not express Atg5 protein in response to CeO_2_ NP (Additional Figure 3). As for their WT counterparts, no major histological modification could be observed 28 days after the administration of 5 μg CeO_2_ NP (Figs. 4 & 5). However, when considering the higher dose of 50 μg CeO_2_ NP, histological analysis of lung tissue sections indicates that *Atg5*^*+/−*^ mice were protected against CeO_2_ NP-induced alveolar fibrosis; no alveolar wall thickening could be observed in these mice (Fig. 4 *: *p* < 0.05). This protection against alveolar remodeling was further confirmed by the quantification of total, type I and III collagen deposition, as well as expression of αSMA and TGF-ß: significant differences between *Atg5*^*+/−*^ and WT mice could be detected for these parameters. In stark contrast, *Atg5*^*+/−*^ mice present the same peribronchiolar fibrosis as compared to their WT counterparts (Fig. 5, *: *p* < 0.05); a significant thickening of bronchiolar walls, together with a significant accumulation of total collagen, collagen I, collagen III, αSMA and TGF-ß in bronchiolar area could be observed in *Atg5*^*+/−*^ mice exposed to 50 μg CeO_2_ NP, and was not different between *Atg5*^*+/−*^ and WT mice.

This protection wasn’t the result of a differential CeO_2_ NP accumulation between WT and Atg5^+/−^ mice, as X-Ray microfluorescence (micro-XRF) showed that the Ce signal could be observed with similar intensity whatever the mice genotype, mainly in the alveolar region, and much less in bronchiolar walls (Additional Figure 4). Moreover, micro X-ray absorption near edge structure (XANES) spectra of Ce spots detected in CeO_2_-exposed mice revealed three different types of modifications that could be observed: 1/ similar to that of the CeO_2_ reference (red line in Additional Figure 4E – Spectrum Type I); 2/ a spectrum presenting a shift of the first peak toward 5727 eV (instead of 5731 eV as in the reference – yellow line – Spectrum Type II), suggesting the reduction of Ce^IV^ to Ce^III^; and 3/ a mixed contribution of Type I and Type II spectra (green line). A similar distribution of the different type of spectra could be observed in all CeO_2_-exposed lungs, whatever the genotype (Table 1).

**Table 1:** Distribution of the different types of XANES spectra in WT and Atg5^+/−^ samples

	Type I	Type II	Mix Type I + II
**WT samples (%)**	36.4	45.4	18.2
**Atg5**^**+/−**^ **samples (%)**	33.3	48.2	18.5

### Characterization of macrophage polarization in response to CeO_2_ NP

**Fig. 6:**
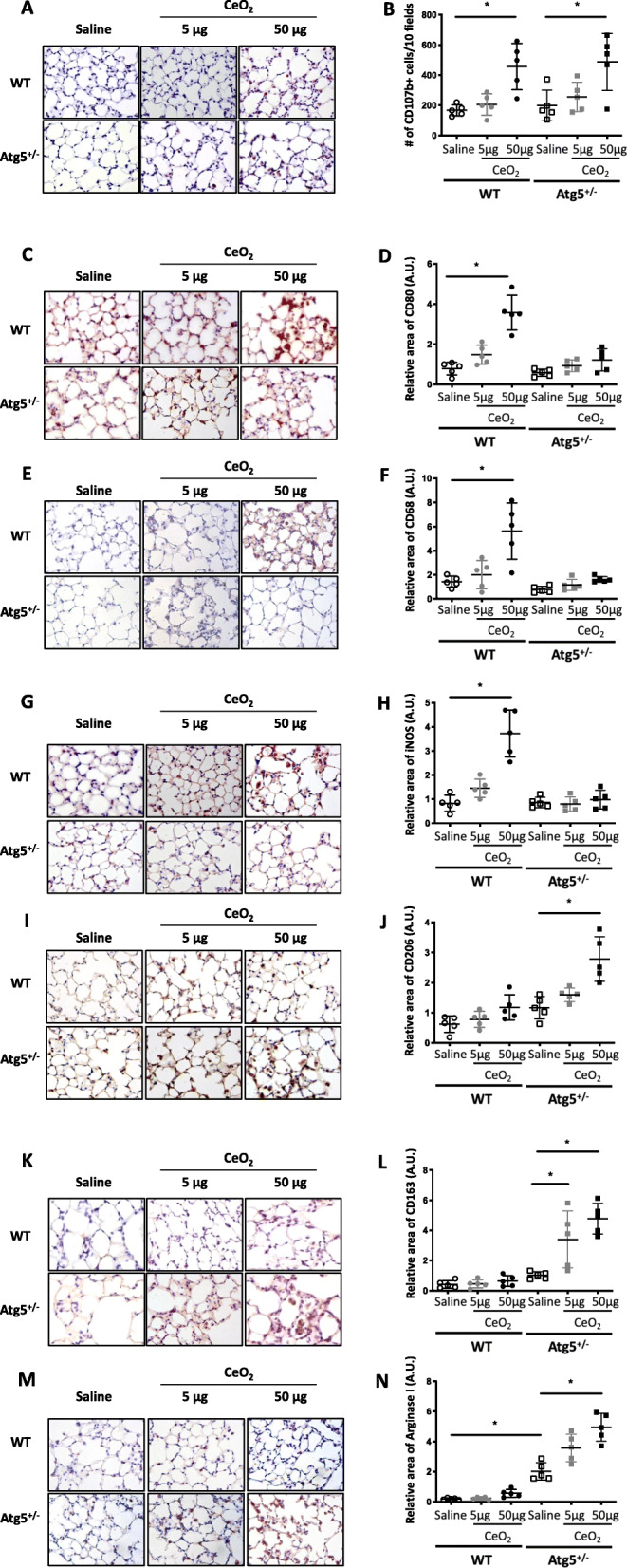
Characterization of CeO_2_-induced macrophage recruitment and polarization in mice. Representative lung tissue sections of C57Bl/6 mice exposed to Saline or CeO_2_ NP (5 or 50 μg, observation at 28 days), after immunostaining for CD107b, as a marker of total number of macrophages (Panel **a**). Original magnification × 200. Quantification of CD107b positive cells (Panel **b**). Representative lung tissue sections of mice exposed to Saline or CeO_2_ NP (5 or 50 μg, observation at 28 days), after immunostaining for CD80 (Panel **c** and **d** for quantification), CD68 (Panel **e** and **f** for quantification), iNOS (Panel **g** and **h** for quantification), CD206 (Panel **i** and **j** for quantification), CD163 (Panel **k** and **l** for quantification) or Arginase 1 (Panel **m** and **n** for quantification). Original magnification × 200. Legends as in Fig. 4

Blockage of macrophagic autophagy didn’t modify the increase in total number of macrophages induced by 50 μg CeO_2_ NP exposure (Fig. 6), while no modification of total macrophage number could be observed in response to 5 μg CeO_2_ NP, whatever the mouse genotype. Indeed, the quantification of CD107b expression in lung tissue sections of CeO_2_-exposed mice, as an index of total number of macrophages in lung tissue, was similar in WT and *Atg5*^*+/−*^ mice, and confirmed the increased total number of macrophages observed in BAL (Fig. 6a and b). However, when assessing the M1 or M2 phenotype of alveolar macrophages as potential contributors to alveolar fibrotic response [15–17], we observed that WT mice exposed to 50 μg CeO_2_ NP (but not 5 μg) present an M1-like phenotype, with significantly increased expression of CD68, CD80 and iNOS proteins as compared to unexposed mice (Fig. 6c-e and i-k, *: *p* < 0.005), whereas *Atg5*^*+/−*^ mice developed an M2-like phenotype, characterized by increased expression of CD206, CD163 and Arginase1 (Fig. 6f-h and l-n,*: *p* < 0.005). These results were confirmed in vitro; exposure of WT peritoneal macrophages to CeO_2_ NP led to the increased expression of M1 markers, while exposure of macrophages obtained from *Atg5*^*+/−*^ mice lead to an increased expression of M2 markers (Additional Figure 5).

**Fig. 7:**
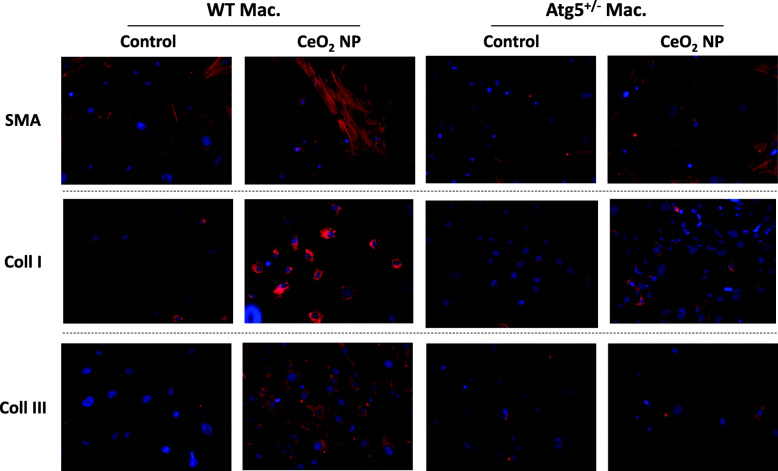
Fibroblast differentiation in vitro. Representative images of WT fibroblasts exposed to supernatant of Vehicle (Control) or CeO_2_ NP-exposed macrophages obtained from WT or Atg5+/− mice, and immuno-stained for SMA, Collagen type I or type III

This modification of macrophage polarization in *Atg5*^*+/−*^ mice could be responsible for the protective effect against the development of alveolar fibrosis in *Atg5*^*+/−*^ mice. Indeed, exposure of WT fibroblasts to the supernatant of CeO_2_-exposed macrophages induced fibroblast-to-myofibroblast differentiation, detected by an increased expression of αSMA, Collagen I and III, only when WT macrophages were used, but not macrophages bearing a *Atg5*^*+/−*^ genotype (Fig. 7).

## Discussion

Taken together, our results show the progressive and dose-dependent development of lung fibrosis in mice exposed to CeO_2_ NP. They also demonstrate that the blockage of macrophagic autophagy protects from alveolar but not bronchiolar fibrosis, probably via the modulation of macrophage polarization in favor of a M2 phenotype.

The progressive and dose-dependent induction of pulmonary fibrosis that we describe here in response to CeO_2_ NP single administration is in accordance with data from literature using other CeO_2_ NP [1, 5–9]. The same is true for the sustained pulmonary inflammation that we detected and which has been shown to be slow to resolve post exposure [19]. We didn’t however observe the formation of granuloma as described for example by Park and colleagues in CeO_2_-exposed animals [8], but this is probably linked to the lower doses of CeO_2_ NP that we used in our study; 5-50 μg per mouse versus 3 mg in Park study. These doses were chosen based on the effects seen in other studies, covering a range from minimal to clearly observed effects. Our results also confirm and extend the data obtained by Ma and colleagues, showing that rats exposure to CeO_2_ NP led to the induction of M1 phenotype in BAL macrophages of these animals [20]. It must be noted however that 28 days after the initial administration, mRNA expression of Arginase 1 was increased in Park study, thus suggesting a shift toward M2 phenotype that was not observed in our WT animals, although care must be taken as only one single M2 marker was targeted in Park’s work.

The development of bronchiolar fibrosis in response to CeO_2_ NP was not prevented by the blockage of macrophagic autophagy. Interestingly, this could be paralleled by the very low Ce elemental signal observed by μXRF in bronchiolar regions, as well as the almost complete lack of macrophages present in these areas, suggesting that CeO_2_-induced bronchiolar fibrosis is independent of macrophages. Although we did not investigate further the biological mechanism(s) underlying this bronchiolar fibrosis, the occurrence of epithelial-mesenchymal transition (EMT) could be an explanation. Indeed, Ma and colleagues have recently demonstrated that exposure to CeO_2_ NP induces EMT in alveolar type II cells that ultimately plays a role in lung fibrosis [5]. Similarly to what we have demonstrated with in vitro exposure of fibroblasts to the supernatant of Ce-exposed macrophages, it could be interesting to explore the effect of the secretome from Ce-exposed epithelial cells on fibroblast to myofibroblast differentiation in our system.

The protection against alveolar fibrogenesis observed in Ce-exposed Atg5^+/−^ mice could have been the result of decreased amounts of Ce present in alveolar regions of Atg5^+/−^ mice. Indeed, autophagy is known to interplay with macrophage phagocytosis [21–23], and M2 polarization has been shown to enhance Si-NP uptake by macrophages [24]. Although we did not strictly quantify the Ce content in WT and Atg5^+/−^ mice exposed to CeO_2_ NP, the similar μXRF signals obtained 28 days after the initial NP administration in both animal genotypes in terms of both intensity and tissue distribution, together with the similar total number of macrophages present in the lungs of WT and Atg5^+/−^ animals strongly suggest that the protection against alveolar fibrosis observed in Atg5^+/−^ animals is probably not the result of a decreased amount of CeO_2_ NP in these individuals. It must be added, however, that the quantification of the initial deposition of NP in the first 24 h after their instillation should deserve further studies in order to fully understand the following fibrotic response. CeO_2_ is highly reactive, and it has been suggested that a change in the Ce^3+^/Ce^4+^ ratio may play a significant role in toxicity determination [25], and could thus contribute to lung fibrosis development. In our experiment, while Ce in CeO_2_ NP was Ce^4+^, Ce in lung tissue was only 33–36% pure Ce^4+^, indicating a change in speciation after NP administration. However, both WT and Atg5^+/−^ mice showed a similar modification of their Ce speciation, with 45–48% as Ce^3+^, and around 18% presenting a mix Ce^3+^/Ce^4+^ form. Therefore, the specific protection against alveolar fibrosis observed in Atg5^+/−^ mice could not be attributed to modifications of Ce speciation.

The modification of macrophage polarization related to their autophagy status could also represent an interesting candidate to explore the underlying mechanism that occurs in Atg5^+/−^ mice [26]. Indeed, macrophages are divided into two distinct sub-populations defined as classically activated pro-inflammatory M1 subtype and alternatively activated M2 subtype responsible for anti-inflammatory, tissue repair and remodeling [27]. Both M1 and M2 macrophages have been noted to be involved in the pathogenesis of pulmonary fibrosis. This highlights the plasticity of macrophage polarization depending on the micro-environment stimuli and signals [28], as well as the fact that assays dedicated to polarization generally capture what happens at a given time point in time and space, as we did in our study, while a wiser approach could have been to consider the timing of the modifications in polarization [29]. In our experimental model of lung fibrosis, we demonstrated that macrophages from autophagy-deficient mice tended to polarize into M2 phenotype whereas their wild-type counterparts exhibiting proficient autophagy skewed towards the M1 subtype. Interestingly, the induction of autophagy by advanced glycation end products or rapamycin triggered macrophage polarization toward M1 phenotype, as well as a sustained inflammation in mice and patients, resulting in delayed wound healing. Moreover, the inhibition of autophagy reduced M1 population, which is in accordance with our results [30]. However, it must be noted that in mice on a high fat diet, there was an increased M1 and decreased M2 polarization in macrophages with defective autophagy, leading to hepatic inflammation and the progression to liver injury [31]. This underlines the importance of the environmental context in the overall autophagy effect, which could be a clue to the effects observed in our study, as to understand the underlying mechanism of macrophage specific polarization in absence of autophagy.

Finally, our results suggest that macrophagic autophagy can facilitate fibrosis. As stated before, the induction of lung fibrosis by CeO_2_ NP is in accordance with experimental data from literature, exploring the consequences of pulmonary administration of CeO_2_ NP but also chemically different NP, such as Silica (Si) NP [1, 5–9, 32]. When it comes to the role of autophagy in this particle-induced lung fibrotic process, the literature is far less clear [33], as it contains both references that autophagy is protective [11, 13, 34, 35], and also that it can be deleterious [36–39]. Using the same mice model, Jessop and colleagues demonstrated a greater silica-induced lung fibrosis, together with enhanced inflammation in Atg5^+/−^ mice as compared to their WT littermates [40]. This result, which is opposite to ours, could be explained by the chemical nature of the particles used (silica versus cerium), together with their size (micro versus nano) as these factors are known to interfere with the resulting effect on autophagy process [41]. Moreover, an important difference between the two studies could be the toxicity which was observed in response to silica but not cerium oxide particles exposure, toxicity that could ultimately lead to the modulation of alternative mechanisms of action linked to fibrosis pathophysiology, such as NLRP3 inflammasome [40, 42], IL-1ß signaling [43, 44], EMT [34], macrophage polarization [38, 45], senescence [37], lipid metabolism [46], or apoptosis [47], or endoplasmic reticulum stress [48]. Recent results from the literature show an increased number of LC3-II puncta, an index of active autophagy, in fibroblast foci of patients with idiopathic pulmonary fibrosis (IPF), the most common form of pulmonary fibrosis [13, 39], which is in accordance with our results, although one can’t know if it results from an effort to protect rather than contribute to the disease. In vitro, these authors also showed that autophagy is induced by TGFß, and that it is necessary for TGFß-induced fibrosis in both non-IPF and IPF fibroblasts [7]. Interestingly, such a cell-specific role for autophagy has been recently described in chronic obstructive pulmonary disease (COPD) where autophagy is believed to be protective except in macrophages [10–12, 34]. In lung fibrosis, the majority of the studies have focused on lung epithelial cells and/or fibroblasts [34], as the two major cell types involved in fibrogenesis [18]. However, the recruitment of inflammatory cells, leading to the activation of effector cells, is often considered as a first trigger of fibrosis [49], and as such, the activation of macrophages occupies a pivotal role in the translation of injury to aberrant repair in lung fibrosis [16, 17]. Interestingly, the LC3 puncta observed by Ghavami and colleagues in fibroblast foci of IPF patients are coherent with the presence of macrophages [39].

Overall, our work provides in vivo as well as in vitro evidences of the role of macrophagic autophagy in CeO_2_ NP-induced lung alveolar fibrosis. We are fully aware that peritoneal macrophages do not fully represent the lung resident macrophages or alveolar macrophages. However, we chose to use them because a large body of literature combining both in vitro approaches using peritoneal macrophages, and in vivo experiments in mice, demonstrates the relevance of the findings obtained in peritoneal macrophages, and their potential translation to what may occur in vivo [50–53]. Moreover, we wanted to be at most in accordance with the 3R’s guidelines, as the number of alveolar macrophages that can be obtained from a broncho-alveolar lavage performed in one single mouse would have required a very high number of animals to obtain a sufficient amount of cells for in vitro experiments. We therefore strongly believe that our in vitro results are relevant to what occurs in vivo, although this specific issue should be addressed by dedicated studies that were beyond the scope of the present study.

## Conclusions

In conclusion, although we are perfectly conscious that one single administration of CeO2 NP doesn’t recapitulate the entire human development of fibrotic disease our findings bring novel insight on the role of macrophagic autophagy in lung fibrogenesis, and add to the current awareness of pulmonary macrophages as important players in the disease.

## Methods

### Experimental model of lung fibrosis

Eight to twelve weeks old C57Bl/6 mice purchased from Janvier (Le Genest-St-Isle, France) were acclimated for 1 week. All mice (maximum 5 per cage) were supplied with food (SAFE, Auguy, France) and tap sterilized water ad libitum in standard wire-topped cages in a controlled environment, with a 12 h light/dark cycle. They then received saline or CeO_2_ NP (5 or 50 μg, NanoAmor, Houston, TX) via non-surgical oropharyngeal instillation (MicroSprayer® Aerosolizer, PennCentury), using the intubation platform and small animal laryngoscope (Penn-Century, Inc) for holding the mice. Before each oropharyngeal instillation, mice have been beforehand anesthetized using intro-peritoneal injection of Ketamine (75 mg/kg) + Xylazine (15 mg/kg) in saline solution. Briefly, stock suspensions of 2 mg/ml NP in 0.9% NaCl were vortexed and bath sonicated for 10 min at 37 kHz just before administration. Each mouse received a volume of 25 μl of stock solution (50 μg dose, or after a 10x dilution in 0.9% NaCl 5 μg dose). CeO_2_ NP used in the present study are from the same supplier and batch as that described in [54]. Shape (spherical) and size (22.4 ± 0.2 nm) were determined by transmission electron microscopy (TEM), crystallinity (cerianite) by X-ray diffractometer, and specific surface area was estimated by Brunauer Emmett Teller analysis (42 ± 0.5 m^2^/g). Moreover, zeta potential (9.5 ± 0.6 mV at pH 7) and hydrodynamic diameter (1480 nm) were determined by dynamic light scattering. No endotoxin content (determined by the Limulus Amebocyte Lysate test) could be detected. Mice were sacrificed 24 h, 1 or 4 weeks later. At that time, each mouse was anesthetized by intraperitoneal injection, with a cocktail of 3.33% buprenorphine, 32.03% zoletil, 4.2% xylazine and 60.43% physiological saline (0.9% saline) at 5 μL/g, and the lungs were harvested and collected for further analysis [54]. A broncho-alveolar lavage (BAL) was performed in a subset of mice as previously described [55]. Briefly, lungs were washed twice with 1 ml of 0.9% saline, and total alveolar cells were collected by a centrifugation at 400 g for 15 min at 4 °C. The resultant cellular pellet was suspended in physiological saline. Cellular viability was assessed as > 90% by trypan blue exclusion and the total number of cells were quantified. For differential counts, the cell suspension was spun (Cytospin-2, Shandon Products Ltd.), fixed in methanol, and stained using Diff Quick solution (Medion Diagnostics, Plaisir, France). Myeloid cell specific *Atg5* deficient mice (Atg5^fl/fl^ LysM-Cre^+/−^ mice further referred as Atg5^+/−^) and their littermate wildtype (WT - Atg^fl/fl^ LysM-Cre^−/−^ − C57Bl/6 background) counterpart were kindly provided by Fatima Clerc [56]. GFP-LC3 mice (C57Bl/6 background) were purchased from Riken, Japan. All mice were subjected to the same procedures. The experimental protocol received the approval of the French Government (Ministère de l’Enseignement Supérieur, de la Recherche et de l’Innovation, APAFIS #14914–2,018,042,515,599,016).

### Histological and immunohistochemistry analyses

Paraffin embedded lung tissue sections (5 μm) were stained with Hematoxylin and Eosin or Sirius Red for histological observations and total collagen deposition, respectively. Immunohistochemistry experiments were performed using antibodies described in Table 2, after a pre-blocking step (incubation of tissue sections with 2.5% horse serum in PBS for 30 min). The duration of the primary antibody incubation was over night at 4 °C. We used biotinylated goat anti-rabbit secondary antibodies for Collagen I, Collagen III, SMA, TGF-ß, CD68, CD80, iNOS, CD163, CD206, Arginase1 (Vector Labs), biotinylated goat anti-rat secondary antibody for CD107b (Vector Labs), horse anti-rabbit IgG (Vector Labs) for Atg5, Alexa Fluor 488 (Green) for LC3 and Alexa Fluor 546 (Red) secondary antibodies for LAMP1 (Invitrogen).

**Table 2:** List of antibodies used in the study

Antibody	Dilution	Reference	Fabricant
**Collagen I**	1:100	AB21286	Abcam, Cambridge, UK
**Collagen III**	1:1000	AB7778	Abcam, Cambridge, UK
**SMA**	1:3000	AB5694	Abcam, Cambridge, UK
**TGF-ß**	1:100	PA5–86215	Thermo-Fisher, France
**CD107b**	1:50	550,292	BD BioScience, France
**CD68**	1:100	AB125212	Abcam, Cambridge, UK
**CD80**	1:100	AB64116	Abcam, Cambridge, UK
**iNOS**	1:100	AB15323	Abcam, Cambridge, UK
**CD163**	1:250	AB182422	Abcam, Cambridge, UK
**CD206**	1:500	AB64693	Abcam, Cambridge, UK
**Arginase-1**	1:500	AB91279	Abcam, Cambridge, UK
**LC3**	1:1000	PM036	MBL International, MA
**LAMP-1**	1:50	AF4320	R&D Systems, France
**Atg-5**	1:400	NB110–53818	Novus Biologicals

At least 10 fields per lung tissue section (magnification 200X) were evaluated for the quantification of histological lesions and immunostainings using ImageJ software as previously described [54]. Briefly, for alveolar wall thickening, alveolar images without bronchi were used to build a macro with threshold value for alveolar wall thickness excluding alveolar spaces. For bronchiolar thickening, at least 10 fields with well defined, large and round shaped bronchi were imaged per animal. The thickness of each individual bronchus was measured manually at four different regions in pixels, and the average pixels calculated from these regions was converted into μm for bronchiolar thickening.

For immunohistological analyses, a representative image of alveolar or bronchiolar regions was opened under ImageJ and zoomed in for 3 colors. The plug-in with color deconvolution was applied on the image followed by choosing color 1 for DAB stain, color 2 for counter-stain, and color 3 for the white background. The next step was adjusting the threshold for 3 color regions and the specific threshold values for each color were noted and close-all option was employed. The image was then opened and plugin-macro-record and plugin-color deconvolution were applied to the image step-wise. Threshold was adjusted, and the noted values for color 2 and color 1 were set for analyzing the measure for each color.

### Primary cells culture

Peritoneal macrophages: Briefly, primary cultures of peritoneal macrophages were obtained as previously described [57]. Briefly, 2 ml of sterile 4% thioglycolate broth (T0157, Sigma-Aldrich, La Verpillère, France) were administered in the peritoneal cavity of C57Bl/6 mice beforehand anesthetized by intraperitoneal injection using a cocktail of 3.33% buprenorphine, 32.03% zoletil, 4.2% xylazine and 60.43% physiological saline (0.9% saline) at 5 μL/g, using a 26G needle. Seventy-two hours later, mice underwent a cervical dislocation, and peritoneal macrophages were harvested from the peritoneal cavity and cultured in DMEM medium, supplemented with 10% fetal bovine serum (FBS) and 1% penicillin/streptomycin. For immunocytochemistry experiments, cells were seeded in 8-well cell culture chamber slides (LabTek, Nunc, ATGC Biotechnology, France) and exposed for 24 h to 10 μg/ml CeO_2_ NP. After incubation with the primary antibody of interest (Table 2), cells were labelled with secondary antibodies: Alexa Fluor 488 (green) for LC3 and Alexa Fluor 546 (red) for LAMP1. The fluorescence images were captured using Zeiss LSM-510 multitracking laser scanning confocal microscope with a Helium/Neon laser at 543 nm and using AxioVision software (Carl Zeiss).

#### Lung fibroblasts

Primary fibroblasts were isolated from C57Bl/6 mouse lungs by mechanic dissection and enzymatic digestion (collagenase 4,1% in HBSS). They were maintained in Dulbecco’s modified Eagle’s medium (DMEM) and Glutamax (Gibco, 31,966–021) containing 10% foetal bovine serum (Eurobio) and streptomycin penicillin (Life Technologies, E1740384 100 μg/ml). Fibroblasts were seeded in 8-well Labtek for 24 h (10 000 cells per well) before being incubated for 48 h with the supernatant of CeO_2_ NP-exposed peritoneal macrophages obtained from WT or Atg5 animals. Immunofluorescence for the protein of interest was then performed as described for peritoneal macrophages, using Alexa Fluor 546 (red).

### X-ray microfluorescence experiments

The localization and speciation of Ce in the lungs were assessed on lung tissue sections embedded in paraffin, using X-ray microfluorescence (micro-XRF) and micro X-ray absorption near edge structure (micro-XANES) respectively, as previously described [54]. Briefly, paraffin-embedded lung tissue sections (10 μm thick) were obtained from WT and Atg5^+/−^ animals, exposed or not to CeO_2_ NP (50 μg, observation at 28 days). Sections were placed between two ultralene foils. These experiments were performed at the LUCIA beamline of the SOLEIL synchrotron (Orsay, France - [58, 59]), at 5.8 keV, just above the Ce L_1_ edge. The beam focalization was assured by a Kirkpatrick Baez (KB) mirror, which allows a beam size of 3.5*2.5 μm^2^ to be reached. The localization of CeO_2_ NP in lung tissue was visualized by mapping the X-ray fluorescence of S. The very efficient flyscan mode was used. Micro-XANES spectra at the Ce L-edge were then collected in areas presenting high S concentrations (attesting of accumulation to lung tissue characteristic of fibrotic lesions). XANES data were obtained after performing standard procedures for pre-edge substraction and normalization using IFEFFIT implemented in the ATHENA® software package [60]. A total of 29 spectra in WT and 22 spectra in Atg5^+/−^ animals have been recorded.

### Statistical analysis

Five to eight mice per experimental group were utilized. Taking into account the possibility of non-normal distribution in the mice population, nonparametric tests (Kruskal–Wallis statistical test followed by Dunn’s multiple comparison test) were used [61]. Values are expressed as the mean ± SEM. Data were analyzed with GraphPad Prism 6.0 (La Jolla, CA) and STATA v13.0 (College Station, TX). For all statistical tests, *p* values smaller than 0.05 were considered as significant.

## Supplementary Information

**Additional file 1: Figure S1.** Cell differential in CeO_2_-exposed mice. Quantification of cell differential in BAL fluid 24 h (Panel A), 1 week (panel B) or 28 days (Panel C) post-exposure to CeO_2_ NP (50 μg, C57Bl/6 mice). Each individual circle represents the value obtained from one animal (empty circle: saline exposure – plain circle: CeO_2_ NP-exposure). *p < 0.05.

**Additional file 2: Figure S2.** Activation of autophagy in vitro. Panel A: Peritoneal macrophages of GPF-LC3 mice exposed to vehicle (Control) or 10 μg/ml CeO_2_ NP (CeO_2_). Panel B: Expression of Atg5 in peritoneal macrophages from C57Bl/6 CeO_2_-exposed mice. Blue color is for DAPI (nucleus) and green is for Atg5. Panel C: colocalization of LC3 (green) and LAMP1 (red) expression in GFP-LC3 mouse peritoneal macrophages in response to vehicle or CeO_2_ NP. Scale bar: 10 μm.

**Additional file 3: Figure S3.** Expression of Atg5 peritoneal macrophages of CeO_2_-exposed mice. Expression of Atg5 in peritoneal macrophages from WT and Atg5^+/−^ mice exposed to 10 μg/ml CeO_2_ NP for 6 h.

**Additional file 4: Figure S4.** X-Ray microfluoresence and XANES spectra of CeO_2_-exposed mice. Representative images of XRF maps of Saline (Panel A and C) and CeO_2_ NP-exposed (Panel B and D) lungs from WT (Panel A and B) or Atg5+/− (Panel C and D) mice (observation at 28 days, 50 μg CeO_2_ NP). Original magnification × 100. False colors used in correlation XRF maps represent P (green), S (blue) and Ce (red). Panel E: XANES spectra at the Ce edge for Reference (blue line), and representative Ce spots (red, green and yellow lines).

**Additional file 5: Figure S5.** Characterization of macrophage polarization in vitro. iNOS (Panel A), CD68 (Panel B), Arginase 1 (Panel C) or CD206 (Panel D) expression in mice peritoneal macrophages in response to Saline or 10 μg/ml CeO_2_ NP for 6 h.

## Data Availability

The datasets used and/or analyzed during the current study are available from the corresponding author on reasonable request.

## Abbreviations

BAL: Broncho-alveolar lavage
CeO_2_: Cerium Oxide
FBS: Fetal Bovine Serum
XANES: X-ray absorption near edge structure
XRF: X-ray microfluorescence
NP: Nanoparticles
REE: Rare earth elements
