# Supplementary material for: Macrophage autophagy protects mice from cerium oxide nanoparticle-induced lung fibrosis
Source: Part Fibre Toxicol. 2021 Feb 1;18:6. doi: 10.1186/s12989-021-00398-y (PMC7852145; doi:10.1186/s12989-021-00398-y)
Supplement: Supplementary file 1 — Additional file 1: Figure S1. Cell differential in CeO2-exposed mice. Quantification of cell differential in BAL fluid 24 h (Panel A), 1 week (panel B) or 28 days (Panel C) post-exposure to CeO2 NP (50 μg, C57Bl/6 mice). Each individual circle represents the value obtained from one animal (empty circle: saline exposure – plain circle: CeO2 NP-exposure). *p < 0.05. [file 12989_2021_398_MOESM1_ESM.pptx]

## Slide 1
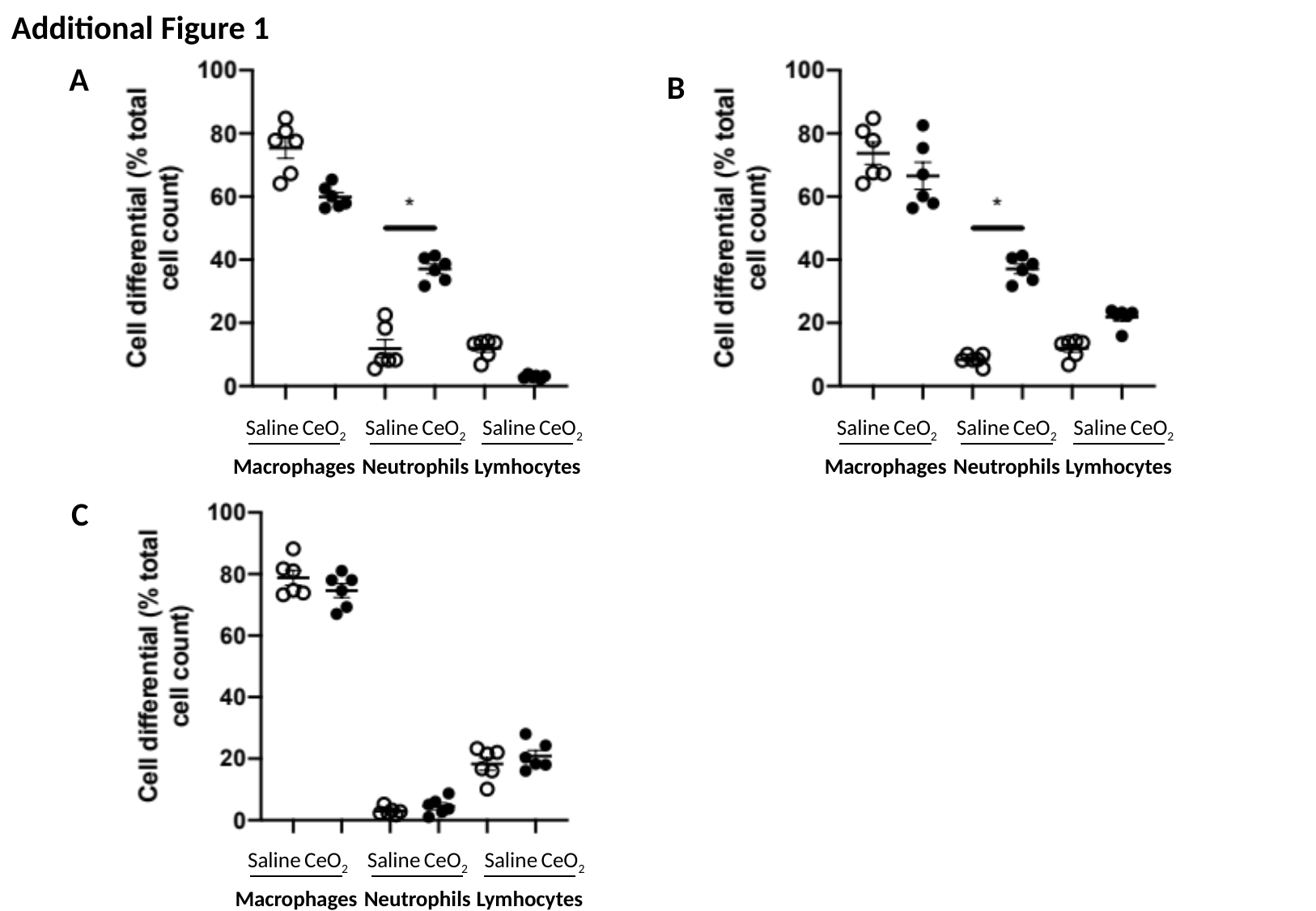

Additional Figure 1
A
Saline
CeO2
Saline
CeO2
Saline
CeO2
Macrophages
Neutrophils
Lymhocytes
B
Saline
CeO2
Saline
CeO2
Saline
CeO2
Macrophages
Neutrophils
Lymhocytes
C
Saline
CeO2
Saline
CeO2
Saline
CeO2
Macrophages
Neutrophils
Lymhocytes
