# Supplementary figures and images for: Macrophage autophagy protects mice from cerium oxide nanoparticle-induced lung fibrosis
Source: Part Fibre Toxicol. 2021 Feb 1;18:6. doi: 10.1186/s12989-021-00398-y (PMC7852145; doi:10.1186/s12989-021-00398-y)

## Slide 1
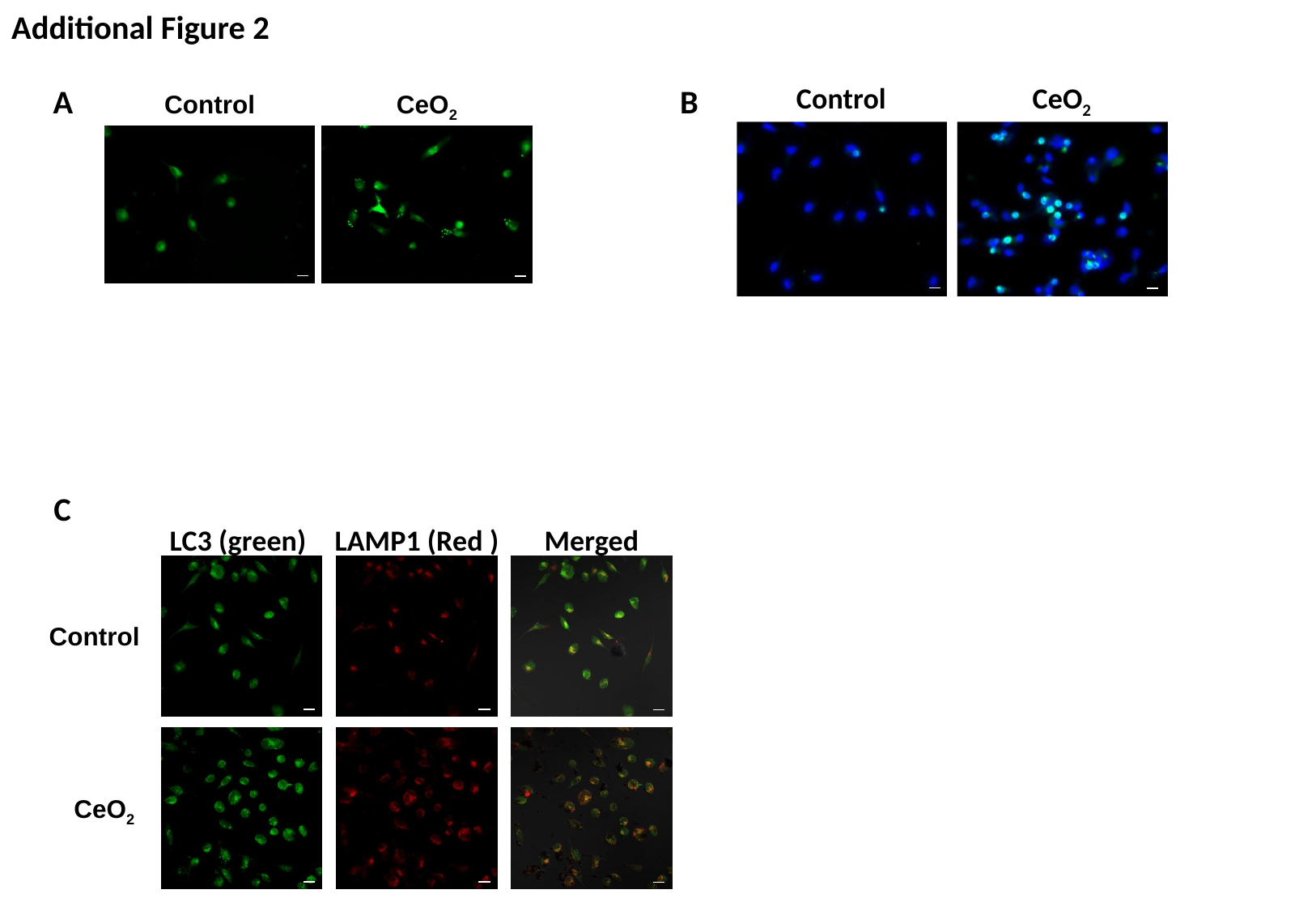

Additional Figure 2
Control
CeO2
A
B
Control
CeO2
C
LAMP1 (Red )
LC3 (green)
Merged
Control
CeO2

Supplement: Supplementary file 2 — Additional file 2: Figure S2. Activation of autophagy in vitro. Panel A: Peritoneal macrophages of GPF-LC3 mice exposed to vehicle (Control) or 10 μg/ml CeO2 NP (CeO2). Panel B: Expression of Atg5 in peritoneal macrophages from C57Bl/6 CeO2-exposed mice. Blue color is for DAPI (nucleus) and green is for Atg5. Panel C: colocalization of LC3 (green) and LAMP1 (red) expression in GFP-LC3 mouse peritoneal macrophages in response to vehicle or CeO2 NP. Scale bar: 10 μm. [file 12989_2021_398_MOESM2_ESM.pptx]

## Slide 1
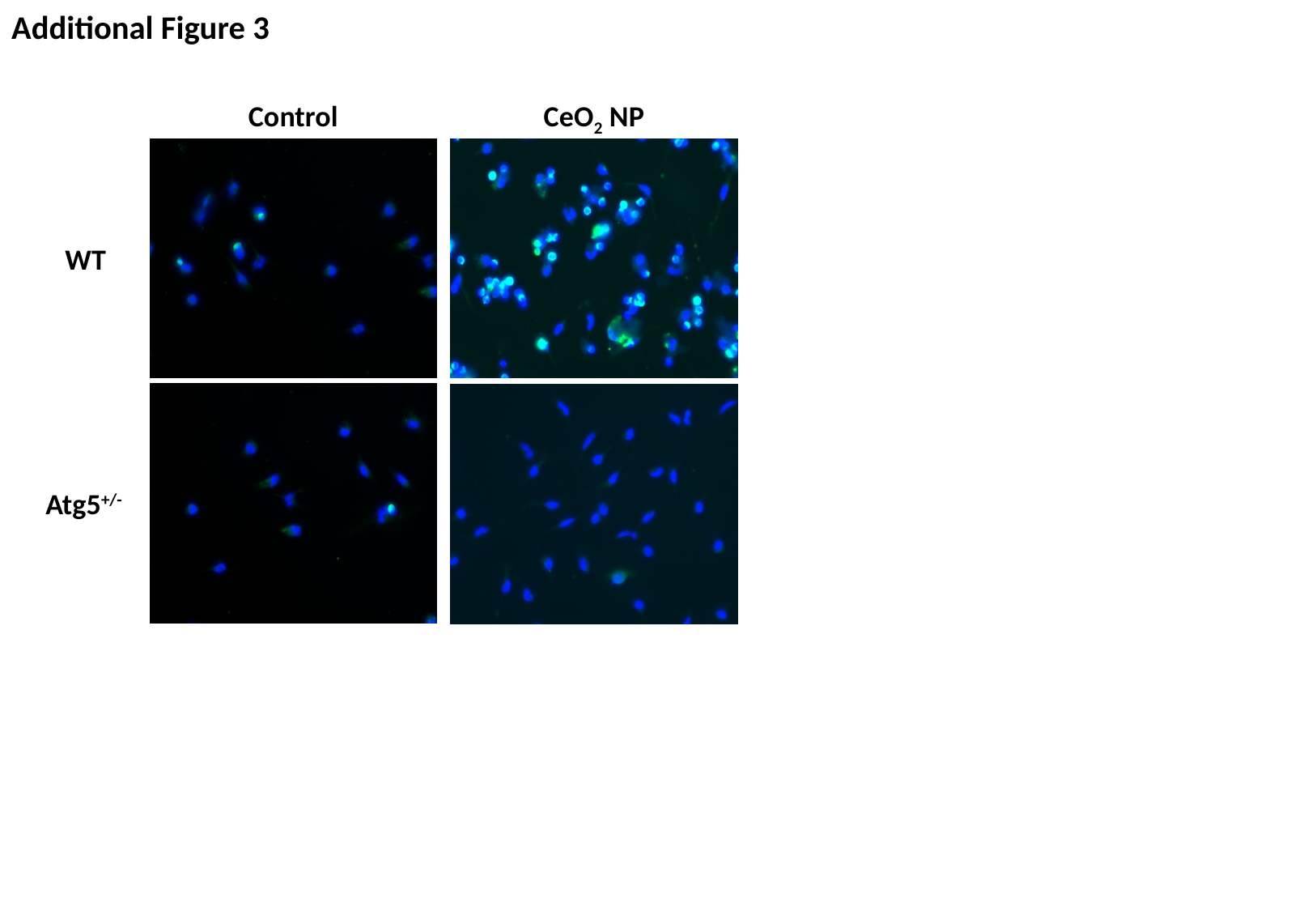

Additional Figure 3
Control
CeO2 NP
WT
Atg5+/-

Supplement: Supplementary file 3 — Additional file 3: Figure S3. Expression of Atg5 peritoneal macrophages of CeO2-exposed mice. Expression of Atg5 in peritoneal macrophages from WT and Atg5+/− mice exposed to 10 μg/ml CeO2 NP for 6 h. [file 12989_2021_398_MOESM3_ESM.pptx]
