# Supplementary material for: Macrophage autophagy protects mice from cerium oxide nanoparticle-induced lung fibrosis
Source: Part Fibre Toxicol. 2021 Feb 1;18:6. doi: 10.1186/s12989-021-00398-y (PMC7852145; doi:10.1186/s12989-021-00398-y)
Supplement: Supplementary file 4 — Additional file 4: Figure S4. X-Ray microfluoresence and XANES spectra of CeO2-exposed mice. Representative images of XRF maps of Saline (Panel A and C) and CeO2 NP-exposed (Panel B and D) lungs from WT (Panel A and B) or Atg5+/− (Panel C and D) mice (observation at 28 days, 50 μg CeO2 NP). Original magnification × 100. False colors used in correlation XRF maps represent P (green), S (blue) and Ce (red). Panel E: XANES spectra at the Ce edge for Reference (blue line), and representative Ce spots (red, green and yellow lines). [file 12989_2021_398_MOESM4_ESM.pptx]

## Slide 1
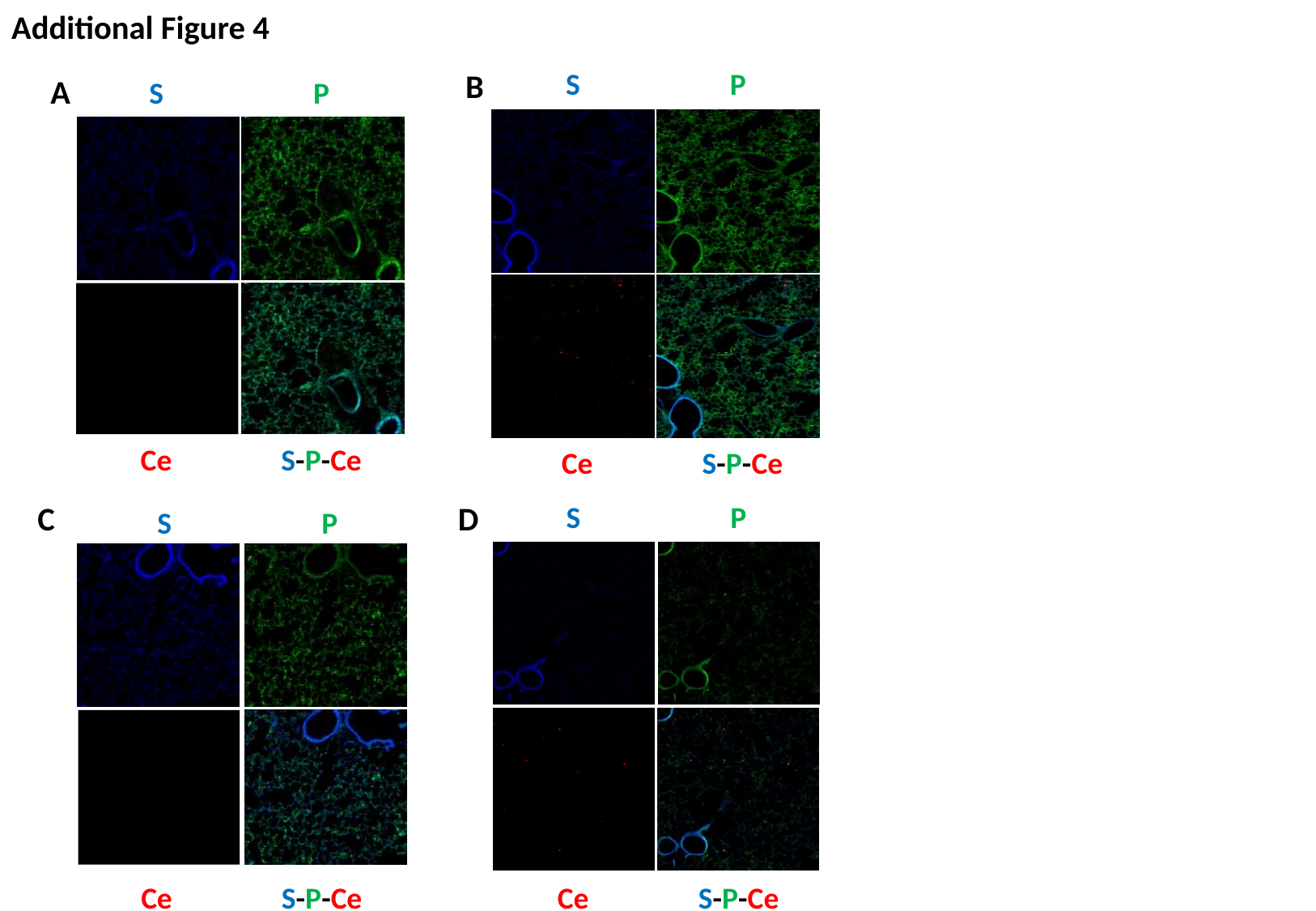

Additional Figure 4
S
P
B
A
S
P
Ce
S-P-Ce
Ce
S-P-Ce
D
S
P
C
S
P
Ce
S-P-Ce
Ce
S-P-Ce

## Slide 2
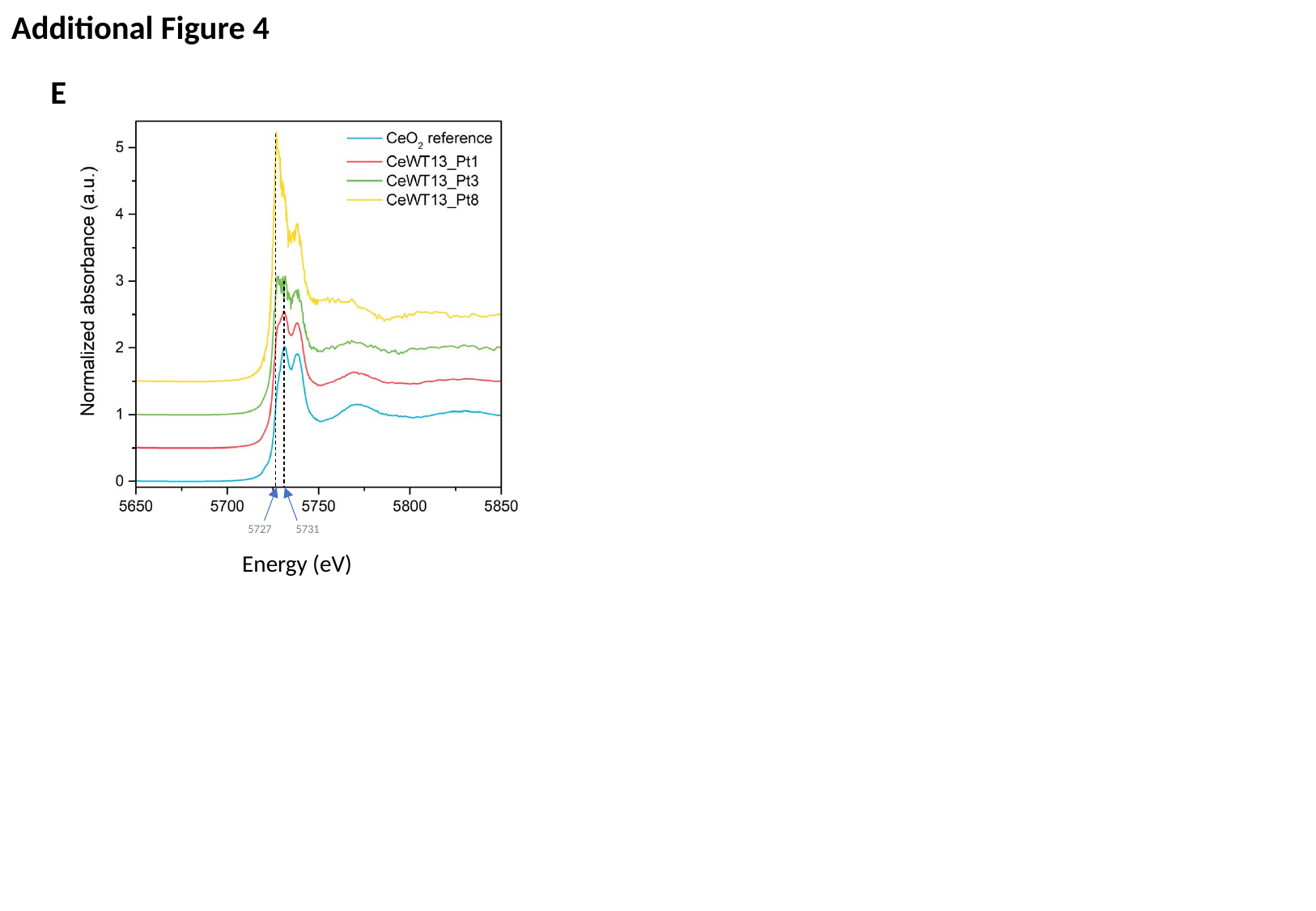

Additional Figure 4
E
5727
5731
Energy (eV)
