# Supplementary material for: Macrophage autophagy protects mice from cerium oxide nanoparticle-induced lung fibrosis
Source: Part Fibre Toxicol. 2021 Feb 1;18:6. doi: 10.1186/s12989-021-00398-y (PMC7852145; doi:10.1186/s12989-021-00398-y)
Supplement: Supplementary file 5 — Additional file 5: Figure S5. Characterization of macrophage polarization in vitro. iNOS (Panel A), CD68 (Panel B), Arginase 1 (Panel C) or CD206 (Panel D) expression in mice peritoneal macrophages in response to Saline or 10 μg/ml CeO2 NP for 6 h. [file 12989_2021_398_MOESM5_ESM.pptx]

## Slide 1
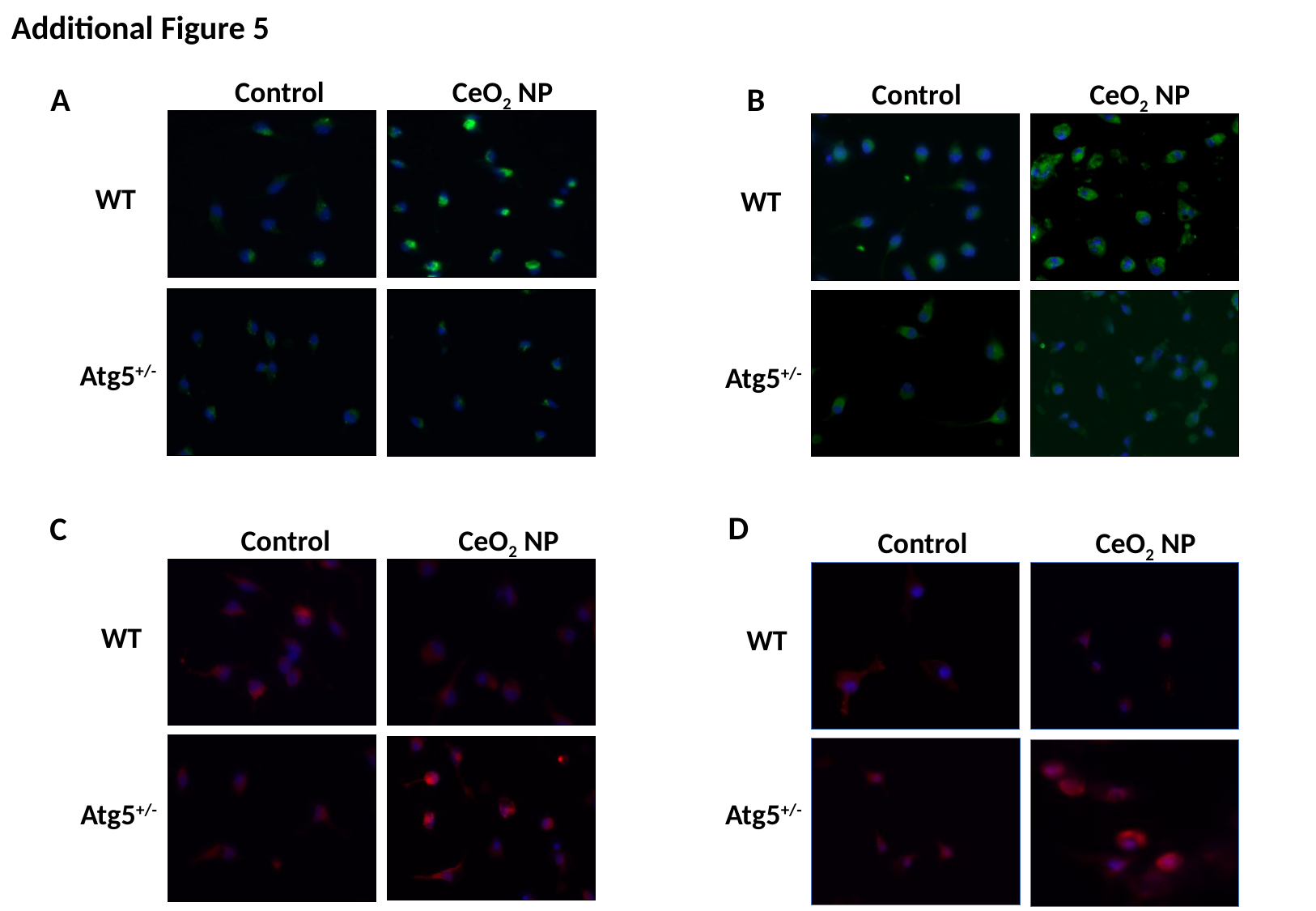

Additional Figure 5
Control
CeO2 NP
Control
CeO2 NP
A
B
WT
WT
Atg5+/-
Atg5+/-
D
C
Control
CeO2 NP
Control
CeO2 NP
WT
WT
Atg5+/-
Atg5+/-

## Slide 2
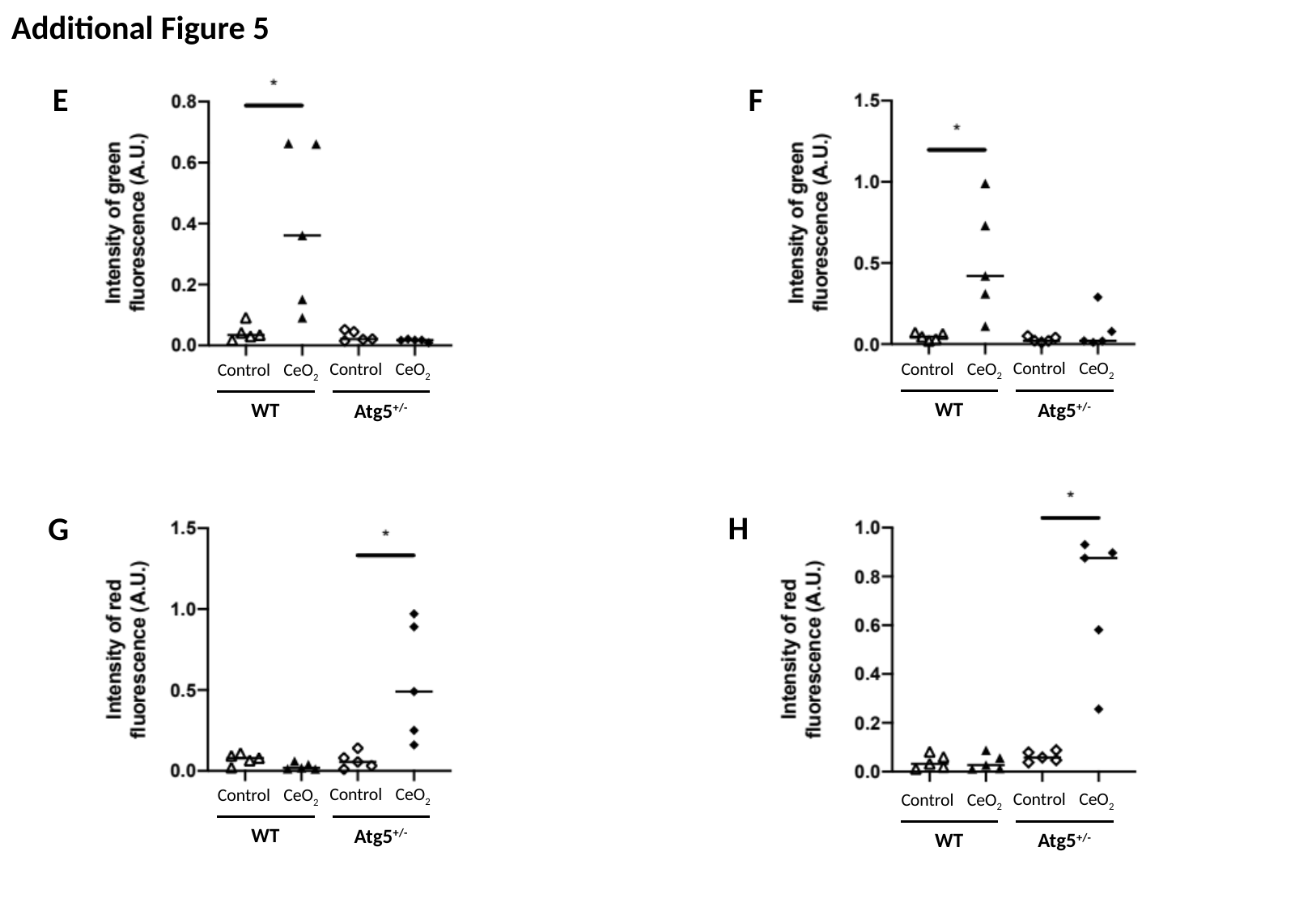

Additional Figure 5
E
F
Control
CeO2
Control
CeO2
WT
Atg5+/-
Control
CeO2
Control
CeO2
WT
Atg5+/-
H
G
Control
CeO2
Control
CeO2
WT
Atg5+/-
Control
CeO2
Control
CeO2
WT
Atg5+/-
